# Processed meat, red meat, white meat, and digestive tract cancers: A two-sample Mendelian randomization study

**DOI:** 10.3389/fnut.2023.1078963

**Published:** 2023-02-13

**Authors:** Zhangjun Yun, Mengdie Nan, Xiao Li, Zhu Liu, Jing Xu, Xiaofeng Du, Qing Dong, Li Hou

**Affiliations:** Department of Hematology and Oncology, Dongzhimen Hospital, Beijing University of Chinese Medicine, Beijing, China

**Keywords:** processed meat, colorectal cancer, Mendelian randomization, red meat, white meat, digestive tract cancers

## Abstract

**Background:**

Previous observational studies suggested inconsistent insights on the associations between meat intake and the risk of digestive tract cancers (DCTs). The causal effect of meat intake on DCTs is unclear.

**Methods:**

Two-sample Mendelian randomization (MR) was performed based on genome-wide association studies (GWAS) summary data from UK Biobank and FinnGen to evaluate the causal effect of meat intake [processed meat, red meat (pork, beef, and lamb), and white meat (poultry)] on DCTs (esophageal, stomach, liver, biliary tract, pancreatic, and colorectal cancers). The causal effects were estimated using a primary analysis that employed inverse-variance weighting (IVW) and complementary analysis that utilized MR-Egger weighted by the median. A sensitivity analysis was conducted using the Cochran Q statistic, a funnel plot, the MR-Egger intercept, and a leave-one-out approach. MR-PRESSO and Radial MR were performed to identify and remove outliers. To demonstrate direct causal effects, multivariable MR (MVMR) was applied. In addition, risk factors were introduced to explore potential mediators of the relationship between exposure and outcome.

**Results:**

The results of the univariable MR analysis indicated that genetically proxied processed meat intake was associated with an increased risk of colorectal cancer [IVW: odds ratio (OR) = 2.12, 95% confidence interval (CI) 1.07–4.19; *P* = 0.031]. The causal effect is consistent in MVMR (OR = 3.85, 95% CI 1.14–13.04; *P* = 0.030) after controlling for the influence of other types of exposure. The body mass index and total cholesterol did not mediate the causal effects described above. There was no evidence to support the causal effects of processed meat intake on other cancers, except for colorectal cancer. Similarly, there is no causal association between red meat, white meat intake, and DCTs.

**Conclusions:**

Our study reported that processed meat intake increases the risk of colorectal cancer rather than other DCTs. No causal relationship was observed between red and white meat intake and DCTs.

## 1. Background

Digestive tract cancers (DTCs) are a severe threat to human health and a substantial economic burden worldwide because of their high morbidity and mortality. In the 2020 Global Cancer Statistics, the top five most common cancers include two DTCs: colorectal cancer (CRC) and stomach cancer (1). Similarly, CRC, liver cancer, and stomach cancer are three of the top five cancers in terms of mortality (1). Studies have revealed that multiple factors mediate DTCs and smoking (2), alcohol consumption (3), obesity (4), and hepatitis B virus infection (5) as potential risk factors for DTCs. Unfortunately, the association between poor dietary habits or nutrition and cancer has received little research attention. Instead, cancer diagnosis and treatment have been the primary focus among scholars. Identifying and eliminating risk factors for cancer is more beneficial to human health than focusing on cancer treatment and diagnosis.

High-fat and high-protein diets have recently become mainstream, and the incidence of CRC has risen from fifth to second from 2018 to 2020 worldwide (1, 6–8). The digestive tract is the primary organ that comes into direct contact with food. Moreover, it is pivotal in the process of food digestion and absorption; therefore, a causal relationship undoubtedly exists between dietary habits and DTCs. Numerous studies have reported a possible correlation between meat intake and DTCs, but the results are inconsistent. For example, a cohort study revealed a negative association between red meat intake and stomach cancer rather than esophageal cancer (9). In contrast, after evaluating and analyzing the quality of 822 published articles about diet and esophageal cancer, Qin and colleagues reported that red and processed meat intake increases the risk of esophageal cancer (10). Similarly, a recent meta-analysis (11) including 400 participants found that red and processed meat intake, but not poultry, was positively associated with CRC risk. However, Mejborn et al. believed that poultry rather than red and processed meat intake increased CRC risk (12). Johnston and colleagues reported that few randomized controlled trials (RCTs) had confirmed the association between red meat and CRC risk (13). Until now, only two RCTs have explored the relationship between red meat and CRC risk. However, both studies presented limited evidence indicating that red meat consumption promoted the risk of CRC (14, 15). In short, cohort or case-control studies have reported contradictory results regarding the associations of meat intake with DTCs. Such inconsistencies may be due to a lack of standardization in study design; moreover, bias and confounding factors cannot be ruled out (16). In addition, the existing observational studies could not establish causality and exclude confounding factors owing to methodological deficiencies, causing bias and disagreements (17). Implementing standard RCTs is difficult because of limitations concerning ethical concerns, time of observation, resources, and cost. Thus, the understanding regarding the causal effect of meat intake on DTCs remains unclear.

Mendelian randomization (MR) studies use single nucleotide polymorphisms (SNPs) that are significantly associated with different types of exposure as instrumental variables (IVs) to assess the association between genetically predicted exposures of interest and outcomes (18). These SNPs are randomly inherited by offspring, providing an analytical approach that simulates an RCT study. As genetic variants before disease onset are randomly assigned at conception, MR studies can rule out confounding factors and prove cause and effect (19).

However, no MR studies explored the potential causal effect of meat intake on DTC risk. Considering that the causal effect of meat intake on DTCs remains unclear, we performed an MR analysis to assess it. This study provided stronger evidence for implementing preventive strategies.

## 2. Materials and methods

### 2.1. Study design

We conducted a two-sample MR based on genome-wide association studies (GWAS) summary data to explore the causal relationship between the intake of five common meat types (processed meat, pork, beef, poultry, and lamb) and six common DTCs (esophageal, stomach, liver, biliary tract, and pancreatic cancers, and CRC). Pork, beef, and lamb are defined as “red meat” and poultry as “white meat.” To avoid sample overlap in exposure and outcome and interference from ethnic differences, we derived GWAS data for both exposure and outcome from the European population but from different cohorts.

### 2.2. IVs for meat intake

**Figure 1:**
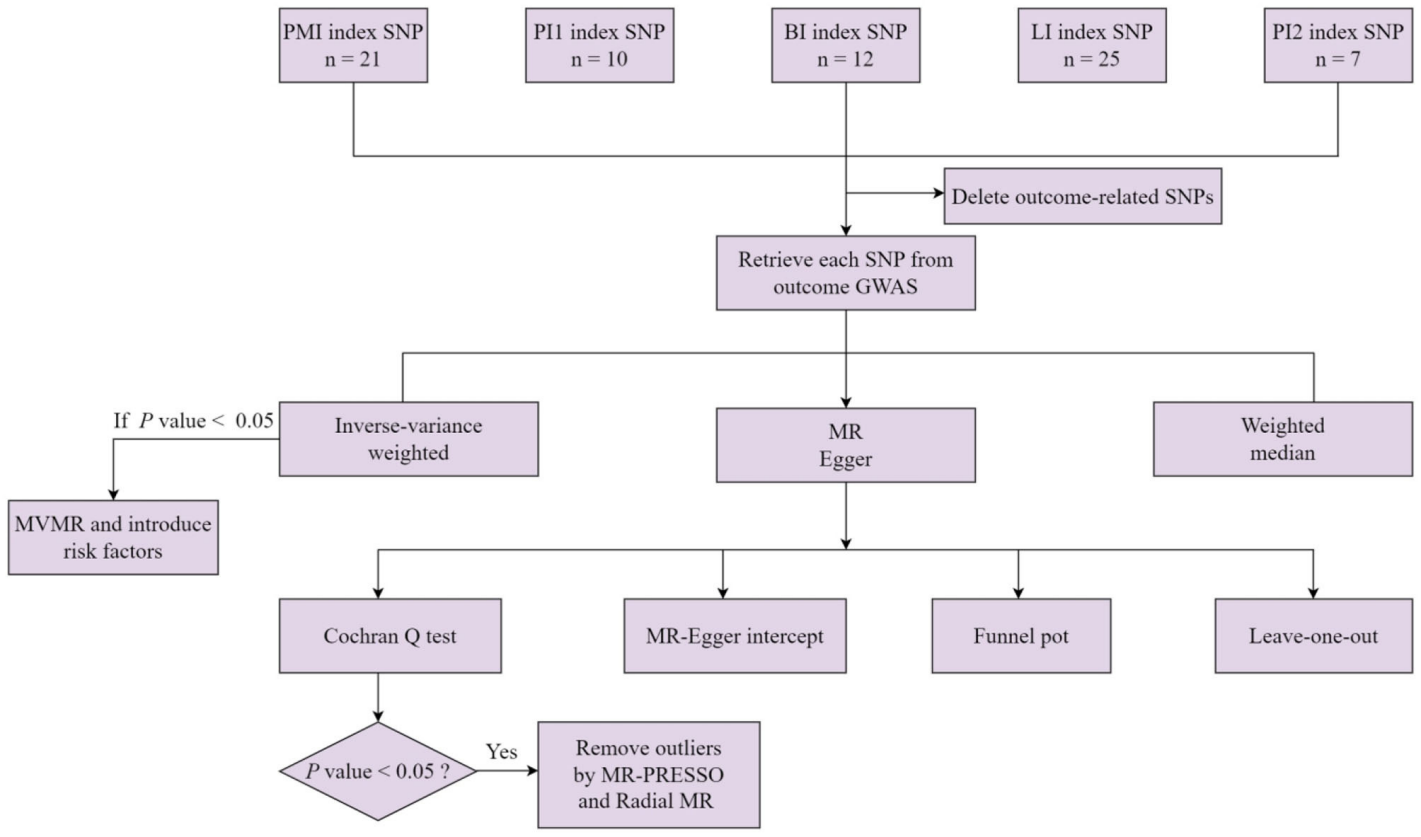
A flowchart for the MR analysis of five types of meat intake and digestive tract cancers. PMI, processed meat intake. PI1, pork intake. BI, beef intake. LI, lamb intake. PI2, poultry intake. MVMR, multivariable Mendelian randomization.

The study process is presented in Figure 1. Summary data for meat intake from the MRC-IEU UK Biobank OpenGWAS (20) based on the study by Elsworth et al. was used as genetic tools for processed meat intake (*n* = 461,981), pork intake (*n* = 460,162), beef intake (*n* = 461,053), lamb intake (*n* = 460,006), and poultry intake (*n* = 461,900). Meat intake was defined by the participants' daily meat intake. All participants were Europeans aged between 40 and 69 years who completed the touchscreen Food Frequency Questionnaire (FFQ) on food intake over the last year (21). Participants had to choose “never” to “once or more daily” for each food intake, and participants with irregular eating habits were excluded (21). For example, how often do you eat processed meats (such as bacon, ham, sausages, meat pies, kebabs, burgers, and chicken nuggets)? Codes include the following: less than one time a week, one time a week, 2–4 times a week, 5–6 times a week, one time or more daily, do not know, and prefer not to answer. The FFQ records detailed data on the frequency of meat intake (https://biobank.ndph.ox.ac.uk/ukb/label.cgi?id=100052). Moreover, a rigorous MR analysis must satisfy three major assumptions: (1) IVs are strongly associated with the exposure of interest; (2) IVs are independent of outcome-relevant confounders; and (3) IVs are not related to the outcome and can only influence the outcome through risk factors (22). We reviewed the SNP quality using rigorous filtering guidelines to satisfy the aforementioned assumptions. To enforce the hypothesis, we set three criteria (23). First, SNPs with *P* < 5 × 10^−8^ were extracted and considered significantly associated with the exposure of interest at the genome-wide level. Second, SNPs were clumped according to the removal of linkage disequilibrium (LD, *R*^2^ > 0.001 and within 10,000 kb). Third, to prevent bias from weak IVs, F statistic values were calculated for each SNP to assess the statistical strength of the IVs. SNPs with *F* < 10 were considered as weak instruments and were removed to ensure that all the SNPs could provide sufficient variation for the corresponding metabolites. To avoid violating hypotheses (2) and (3), the interference of potential confounders and horizontal pleiotropy were excluded (IVs affect outcomes through other exposures rather than the exposure of interest). Thus, each SNP in IVs was examined using a PhenoScanner (www.phenoscanner.medschl.cam.ac.uk), which documented the details of SNP-related genotypes and phenotypes. Furthermore, SNPs associated with potential confounders and outcomes of genome-wide significance were deleted. The MR-Egger method was performed to examine the presence of horizontal pleiotropy in the results (23). Finally, any ambiguous or palindromic SNPs were removed to ensure the consistency of alleles between the exposure and outcome.

### 2.3. GWAS summary data for DTCs

GWAS data related to the six DTCs—including esophageal cancer (cases = 410), stomach cancer (cases = 1,054), liver cancer (cases = 518), biliary tract cancer (cases = 187), pancreatic cancer (cases = 1,054), and CRC (cases = 4,957)—were accessed from the Finngen database (https://www.finngen.fi/en/access_results) (24) R7 release on 19 September 2022. The Finngen study cohort included 309,154 participants, after the exclusion of those with indeterminate sex, high genotype deficiency (>5%), excess heterozygosity (±4 SD), and non-Finnish ancestry (24). Cancer was diagnosed based on the International Classification of Disease codes (8th, 9th, and 10th revisions).

### 2.4. Multivariable MR and risk factors

The univariable MR analysis provided compelling evidence for a causal relationship between genetically proxied processed meat intake and CRC. To confirm the actual association between processed meat intake and CRC, we performed reverse MR and multivariable MR (MVMR) analyses. The reverse MR analysis confirmed the absence of a causal effect between the exposure of interest and the outcome. MVMR analysis assesses the direct effect of the exposure of interest on outcomes by controlling for potential effects between exposures (25, 26). MVMR analysis can prove that processed meat intake can directly affect CRC, independent of other meat intakes. This study performed an MVMR analysis using the multivariable random-effects multiplicative inverse variance weighted method. Moreover, to further explore the potential mechanism through which processed meat intake increases CRC risk, we used mediating variables, such as BMI and total cholesterol (TC), in the analysis. These are widely recognized risk factors for CRC and have been confirmed by previous MR studies for their causal effect on CRC (27, 28). The GWAS data for BMI and TC were obtained from the Genetic Investigation of Anthropometric Traits (29) consortium and the UK Biobank (30), respectively. To assess whether the BMI or TC could mediate the causal effect of processed meat intake on CRC, processed meat intake was considered exposure, and BMI and TC were considered outcomes while performing the MR analysis. Details of all GWAS data for exposure and outcome are presented in Table 1.

**Table 1:** Details of all GWAS data in this study.

**Phenotype**	**Consortium**	**Sample size**	**Ancestry**	**GWAS ID**
Processed meat intake	MRC-IEU	461,981	European	ukb-b-6324
Pork intake	MRC-IEU	460,162	European	ukb-b-5640
Beef intake	MRC-IEU	461,053	European	ukb-b-2862
Lamb intake	MRC-IEU	460,006	European	ukb-b-14179
Poultry intake	MRC-IEU	461,900	European	ukb-b-8006
Esophageal cancer	Finngen	239,088	European	NA
Stomach cancer	Finngen	239,732	European	NA
Liver cancer	Finngen	239,196	European	NA
Biliary tract cancer	Finngen	238,865	European	N/A
Pancreatic cancer	Finngen	239,732	European	NA
Colorectal cancer	Finngen	243,635	European	NA
Body mass index	GIANT	322,154	European	NA
Total cholesterol	UK Biobank	441,016	European	NA

### 2.5. Statistical analysis

For a more comprehensive assessment of the causal effect of meat intake on DTCs, we performed an MR analysis using random-effect inverse-variance weighted (IVW), MR-Egger, and weighted median. The aforementioned approaches are based on different assumptions; however, each approach has its own advantages. Our estimates are primarily based on IVW analysis because IVW is under the hypothesis that horizontal pleiotropy is absent for all SNPs, and IVW provides the most accurate assessment under the following premise (31). Moreover, other MR methods, such as the MR-Egger method and the weighted median, were complementary to IVW to more comprehensively assess the causal relationship between exposure and outcome. Both methods offer a more robust analysis under more generous parameters. The weighted median model allows at least 50% of the SNPs to have no pleiotropy and is affected by outliers to a lesser extent (31). The MR-Egger model allows for pleiotropy in all genetic instruments, detects horizontal pleiotropy, and allows for greater heterogeneity (32). Horizontal pleiotropy occurs when exposure-related IVs directly affect outcomes through pathways other than the exposure of interest. To evaluate the robustness and potential biases of our results, we conducted a sensitivity analysis using multiple methods. These methods included the Cochran *Q* statistic, the MR-PRESSO test, Radial MR, the funnel plot, the MR-Egger intercept, and leave-one-out (LOO) analyses (32, 33). We first identified any possible heterogeneity in our results by calculating the *P*-value from the Cochran *Q* test. We then looked for outliers that may have been affected by pleiotropic bias and removed them using MR-PRESSO and Radial MR (33, 34). Funnel plots were used to check for any bias in the direction of pleiotropy. We also evaluated horizontal pleiotropy by determining the *P*-value of the MR-Egger intercept.

### 2.6. Ethical consideration

All data in this study are available in publicly available databases. No additional ethical approval was required.

## 3. Results

Following the rigorous selection criteria, 21, 10, 12, 25, and 7 SNPs were identified to genetically predict the intake of meat, pork, beef, lamb, and poultry, respectively (Supplementary Table S1). The F statistic values for all of the genetic instruments used in the study were >10, indicating their high quality and reliability. The primary results of the MR analysis were determined based on IVW analysis results. Our findings did not support the causal effect between genetically predicted pork, beef, poultry, and lamb intake and esophageal cancer, stomach cancer, liver cancer, biliary tract cancer, pancreatic cancer, and CRC, with an IVW-derived *P*-value of >0.05 (Table 2). Surprisingly, we found that only the intake of processed meat had a significant causal effect on CRC (IVW: *P* < 0.05), but not on esophageal cancer, stomach cancer, liver cancer, biliary tract cancer, or pancreatic cancer (IVW: *P* > 0.05) (Table 2). However, heterogeneity was only detected in the MR analysis of pork intake and CRC, with a Cochran Q test-derived *P*-value of 0.02. Two outliers (rs2387807, rs3964074) were identified using the MR-PRESSO and Radial MR methods (Supplementary Figure S1). With the deletion of these two outliers and re-application of the MR analysis, heterogeneity became insignificant (Cochran *Q* test-derived *P*-value = 0.24). Heterogeneity (Cochran Q test-derived *P*-value > 0.05) and horizontal pleiotropy (MR-Egger intercept-derived *P*-value > 0.05) were not detected in any of the other MR analysis results (Table 2). The results of the MR-Egger and weighted median analyses are presented in Supplementary Table S2. Scatter and symmetric funnel plots revealed the absence of pleiotropic bias. The LOO analysis revealed that our estimation results are robust. All scatter plots, funnel plots, and LOO plots are displayed in Supplementary Figures S2–S8.

**Table 2:** IVW analysis results for five meat intakes and six digestive tract cancers.

**Exposures**	**Outcomes**	**No. SNPs**	**OR (95% CI)**	***P-*value**	**Heterogeneity**	**Pleiotropy**
Processed meat intake	OC	21	2.36 (0.20–27.55)	0.493	0.31	0.32
Pork intake	OC	10	13.09 (0.11–1,605.65)	0.295	0.71	0.34
Beef intake	OC	12	2.39 (0.03–167.21)	0.688	0.24	0.73
Poultry intake	OC	7	0.36 (0.00–63.96)	0.696	0.74	0.24
Lamb intake	OC	25	0.53 (0.03–10.71)	0.678	0.44	0.42
Processed meat intake	SC	21	1.78 (0.42–7.56)	0.434	0.65	0.35
Pork intake	SC	10	0.21 (0.00–9.18)	0.415	0.11	0.37
Beef intake	SC	12	0.94 (0.05–16.71)	0.968	0.13	0.26
Poultry intake	SC	7	12.44 (0.46–332.65)	0.133	0.41	0.19
Lamb intake	SC	25	2.21 (0.26–19.03)	0.470	0.13	0.83
Processed meat intake	LC	21	0.20 (0.03–1.58)	0.127	0.97	0.43
Pork intake	LC	10	9.89 (0.14–722.95)	0.295	0.72	0.78
Beef intake	LC	12	5.61 (0.19–164.18)	0.316	0.54	0.20
Poultry intake	LC	7	0.18 (0.00–96.90)	0.595	0.09	0.51
Lamb intake	LC	25	0.21 (0.01–4.10)	0.304	0.06	0.06
Processed meat intake	BTC	21	2.23 (0.05–97.41)	0.678	0.22	0.99
Pork intake	BTC	10	0.02 (0.00–273.48)	0.432	0.07	0.86
Beef intake	BTC	12	0.13 (0.00–35.42)	0.478	0.80	0.55
Poultry intake	BTC	7	0.30 (0.00–1,535.78)	0.782	0.29	0.60
Lamb intake	BTC	25	0.16 (0.00–20.86)	0.456	0.20	0.97
Processed meat intake	PC	21	0.82 (0.19–3.48)	0.787	0.60	0.14
Pork intake	PC	10	0.19 (0.00–3.91)	0.283	0.77	0.38
Beef intake	PC	12	0.34 (0.02–4.56)	0.412	0.27	0.53
Poultry intake	PC	7	6.34 (0.24–164.80)	0.267	0.55	0.55
Lamb intake	PC	25	1.32 (0.15–11.95)	0.804	0.10	0.52
Processed meat intake	CRC	21	2.12 (1.07–4.19)	0.031	0.49	0.38
Pork intake	CRC	8	0.28 (0.04–1.82)	0.181	0.24	0.63
Beef intake	CRC	12	1.15 (0.32–4.16)	0.837	0.20	0.57
Poultry intake	CRC	7	0.27 (0.04–1.61)	0.149	0.22	0.16
Lamb intake	CRC	25	0.99 (0.36–2.72)	0.987	0.14	0.22

No. SNPs: the number of SNPs in the MR analysis; P-value: IVW-derived P: P-value of <0.05 represents the causal effect between exposure and outcome: heterogeneity and Cochran's Q-derived P-value; Pleiotropy and MR-Egger intercept-derived P-value. OC, esophageal cancer; SC, stomach cancer; LC, liver cancer; BTC, biliary tract cancer; PC, pancreatic cancer; CRC, colorectal cancer.

**Figure 2:**
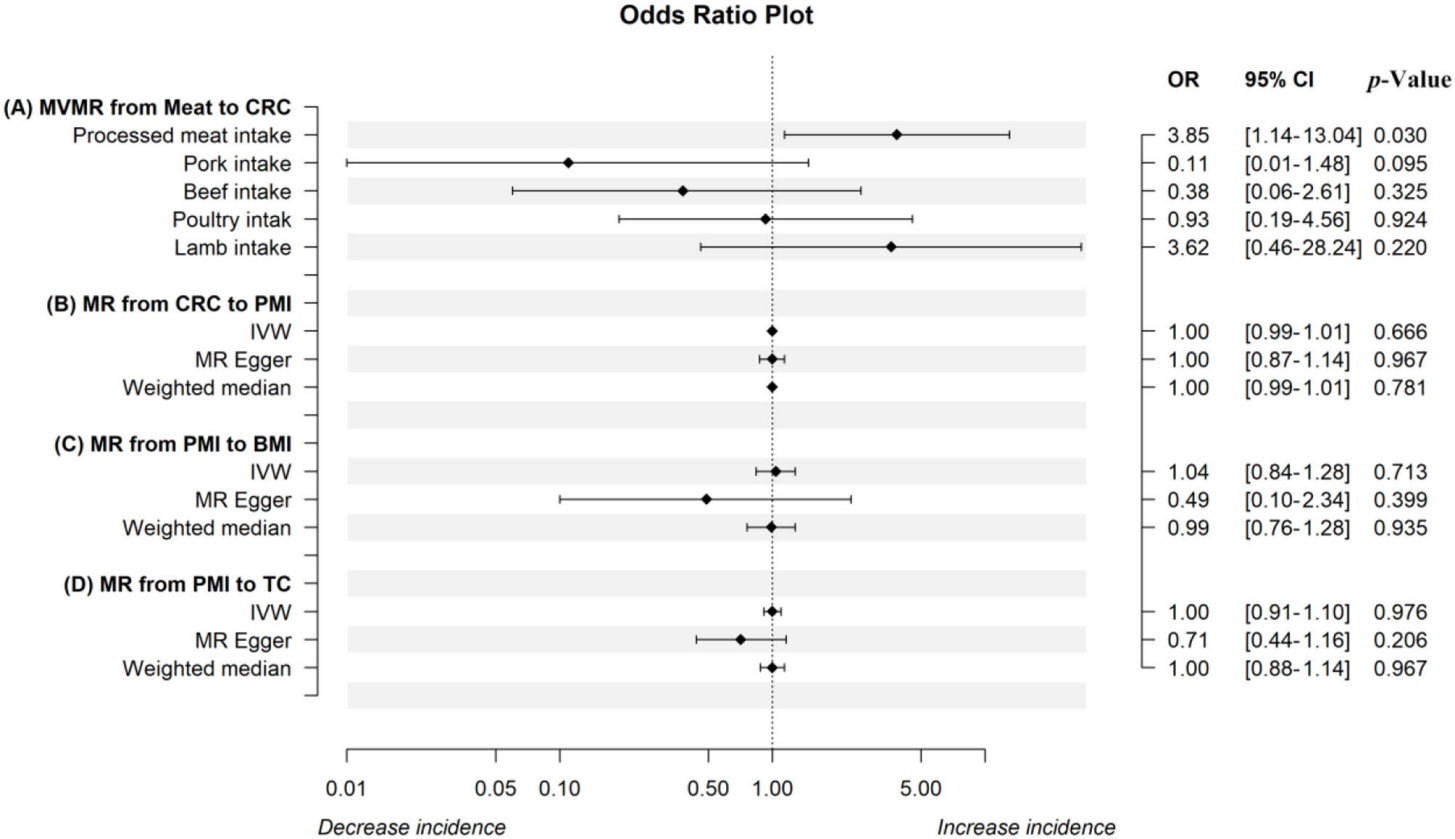
The odds ratio plot of the MR analysis. The odds ratio plot (A) shows estimates of the MVMR analysis from meat intake on CRC in IVW methods when controlling for the other four factors, respectively. The results from IVW, the MR-Egger method, and the weighted median in the univariable MR analysis from CRC to PMI, PMI to BMI, and PMI to TC were displayed in odds ratio plots (B–D). MR, mendelian randomization; MVMR, multivariable Mendelian randomization; IVW, inverse-variance weighted; CRC, colorectal cancer; PMI, processed meat intake; BMI, body mass index; TC, total cholesterol.

The IVW analysis revealed that genetically predicted processed meat intake can significantly promote CRC risk [odds ratio (OR) = 2.12, 95% confidence interval (CI) 1.07–4.19; *P* = 0.031] (Table 2). Each standard deviation (SD) increase in genetically predicted processed meat intake enhanced CRC risk by 112%, according to the IVW analysis. However, the MR–Egger (OR = 9.53, 95% CI 0.34–269.39; *P* = 0.202) and weighted median (OR = 2.51, 95% CI 0.26–24.55; *P* = 0.428) analyses revealed a consistent direction, but the results were not significant. As mentioned earlier, the three analysis methods were established based on different assumptions, which resulted in inconsistent estimates. However, the IVW analysis results are widely acknowledged to be the most accurate. Meanwhile, the consistency of their directions is not accidental, improving our results' persuasiveness. Moreover, there is no evidence of heterogeneity in the MR analysis results because the Cochran *Q* test-derived *P*-value was 0.49. Similarly, the MR–Egger intercept outcome exhibited no horizontal pleiotropy (*P* = 0.38). Scatter plots did not present significant intercepts, and funnel plots were symmetrical, demonstrating that the results were not heterogeneous or pleiotropic (Supplementary Figures S9A, C). The LOO analysis results suggested that rs4240672, rs203319, rs6765179, rs9809856, rs6786550, and rs2029401 could potentially impact the IVW analysis results (Supplementary Figure S9B). The MVMR analysis also proved that processed meat intake could directly affect CRC without interference from other exposures of interest [OR = 3.85, 95% CI 1.14–13.04; *P* = 0.030 (Figure 2A)]. Then, we performed a reverse MR analysis, considering CRC as the exposure and processed meat intake as the outcome. No evidence supports the hypothesis that genetically related CRCs can influence processed meat intake (IVW: OR = 1.00, 95% CI 0.99–1.01; *P* = 0.666) (Figure 2B). As shown in Supplementary Figures S10A–C, the results were not heterogeneous or pleiotropic (*P*-value of heterogeneity = 0.99; *P-*value of pleiotropy = 0.99). Thus, no reverse causality existed between the exposure of interest and the outcome.

To further investigate the potential mechanism through which genetically established processed meat intake increases CRC risk, we introduced the mediating variables BMI and TC to explore whether the aforementioned common risk factors violate the causal effect. The Cochran Q test-derived *P*-values of BMI and TC were 2.752078 × 10^−28^ and 8.779711 × 10^−46^, which indicated that the results were heterogeneous. Through the MR-PRESSO and Radial MR analyses, 8 (rs11887120, rs1422192, rs203319, rs2873054, rs4077924, rs4778053, rs7531118, and rs838133) and 10 outliers (rs10454812, rs1422192, rs2873054, rs4240672, rs4778053, rs6010651, rs6786550, rs6961970, rs77165542, and rs838133) were identified, respectively (Supplementary Figures S11A, E). After these outliers were removed, the MR analysis was re-performed. However, evidence supporting a causal relationship between processed meat intake and BMI and TC is limited (Figures 2C, D). Scatter and funnel plots indicate the absence of heterogeneity and pleiotropy (Supplementary Figures S11B, C, F, G). The LOO sensitivity analysis proved the robustness of the results (Supplementary Figures S11D, H).

## 4. Discussion

We used multiple MR methods to analyze large-scale GWAS data from the MRC-IEU UK Biobank OpenGWAS and Finngen to investigate the causal effect of genetically proxied meat intake on DTCs. The univariable MR analysis only indicated the negative causal effect of processed meat intake on CRC. This estimate is consistent with that made in the MVMR analysis after multiple corrections for other exposures. BMI and TC do not appear to be potential mediators. Our findings do not support associations between DTCs and other exposure of interest. Evidence indicating the results of the MR analysis with pleiotropy and heterogeneity bias is scarce. Furthermore, observational studies produced controversial conclusions because of the unavoidable interference of confounding factors and reverse causation. However, the MR analysis results positively support the causal effect of processed meat rather than red or white meat intake on CRC, except for a bias due to a more rigorous MR design.

Despite the contradictory conclusions, observational studies provided evidence that meat intake is associated with cancer risk (35). The rapid increase in CRC prevalence and CRC-associated mortality is primarily due to changing diet structures (36). By constructing a risk model, Parra-Soto and colleagues analyzed the associations between diet and cancer in a study involving a cohort of 409,110 participants with a mean follow-up period of 10.6 years. They revealed that processed meat intake increases the risk of CRC and prostate cancers (37). Another prospective study of 472,377 UK Biobank participants with a median follow-up of 1.4 years found that people who ate less red meat were less likely to develop CRC and breast cancer (38). Another large prospective study also concluded that consumption of red and processed meat, and not poultry, promoted CRC risk (hazard ratio = 1.35, 95% CI 0.96–1.88) (39). Moreover, a meta-analysis of 60 case-control or cohort studies published between 2016 and 2017 found that processed meat consumption increased the risk of colon cancer, but not rectal cancer (40). RCTs provide more compelling evidence compared with case-control and cohort studies. Until now, only two RCTs explored the associations between red meat (no processed meat) and CRC (14, 15). The results of these RCTs provided robust evidence that red meat intake has no bearing on CRC risk, which is consistent with the results of our study. Therefore, a causal effect of genetically predicted processed meat, but not red meat, is believed to exist on CRC.

However, the potential mechanisms through which processed meat increases CRC risk are poorly defined and may be mediated by the following pathways. Multiple studies confirmed that cancerogenic substances such as polycyclic aromatic hydrocarbons (PAHs), heterocyclic amines (HCAs), N-nitroso compounds (NOCs), and heme iron are formed when meat is processed, such as fried or grilled, at a high temperature for a long time. Creatine and creatinine in meat produce HCAs during high-temperature processing, and more HCAs are produced with an increase in temperature and time (41). Approximately 25 HCAs have been identified and classified into aminoimidazo-azarenes, and carbolines or pyrolytic HCAs. The aforementioned HCAs are metabolically activated to induce DNA sequence mutations and promote cell proliferation, thereby leading to cancer (42). Cytochrome P450 (CYPs) enzymes, CYP1A1 and CYP1B1, induce PAHs, covalently binding to DNA to promote DNA sequence variation (43).

Furthermore, not only can high-temperature processing produce NOCs but the heme iron present in meat can also induce the endogenous synthesis of NOCs and genotoxic free radicals (44, 45). In summary, the aforementioned carcinogenic substances interact with DNA, resulting in genetic mutations that promote cancer development. Therefore, we hypothesize that the causal effect of genetically proxied processed meat intake on CRC may be mediated by the cancerogenic substances produced during meat cooking.

In addition, we introduced two obesity-associated phenotypes, such as BMI and TC, to further explore the potential mediators of the causal effects between processed meat and CRC. Strong evidence confirming that chronic inflammation and sex hormone metabolism mediate obesity and cancer is available, with moderate evidence supporting the role of insulin and IGF signaling (46). Because of the high metabolic activity of adipose tissue, pro-inflammatory factors such as interleukin (IL)-6 and tumor necrosis factor (TNF)-alpha secreted by this tissue can initiate tumor formation (47). Inflammation from the adipose tissue causes insulin resistance, and insulin triggers cancer through antiapoptotic effects (48, 49). Moreover, the pro-cancer effects of TC are widely accepted to be mediated through multiple mechanisms, including TC-induced NLRP3 inflammasome activation (50). However, our findings suggest that the association between processed meat and CRC is independent of BMI and TC. This may be due to the formation of cancerogenic substances due to fatty tissue cleavage during the meat cooking process.

Our findings do not support the evidence that the causal effect of meat intake on DTCs is none other than that of processed meat intake on CRC. A previous meta-analysis including 4 cohorts and 31 case-control studies concluded that processed and red meat increases esophageal cancer risk (51). However, a case-control study presented contradictory results; meat intake was unrelated to esophageal cancer in that study (52). This inconsistent finding is also observed in other DTCs. As Händel et al. reported, the relationship between processed meat and DCTs varied considerably between cohort and case-control studies (16). Risk estimates were higher in case-control studies because these studies had more confounding factors. Until now, standard, large-scale RCTs for verifying their true relationship were scarce.

Our research has the following advantages. To the best of our knowledge, this study conducted the first MR analysis to explore the causal effects of five common meat intakes on DCTs. The greatest strength of our study is the detection of causal effects through minimal confounding. The consistency in the results of univariable and multivariable MR analyses reinforces the evidence that consumption of genetically proxied processed meat, rather than red and white meat, promotes CRC risk. However, our study has some limitations. This study is based on European populations, and how well the findings fit in other populations remains unclear. Moreover, the moderate relationship between cancer and meat intake may have been overlooked because of the low number of cases.

In summary, processed meat intake can directly increase CRC risk, independent of whether red or white meat has been consumed. Advocating for a reduction in processed meat intake is beneficial for the early prevention of CRC. Previous findings have violated the real association due to interference from confounding factors and reverse causation.

## 5. Conclusions

Our study revealed that processed meat intake increases the risk of CRC rather than other DCTs. No causal relationship was observed between red and white meat intake and DCTs.

## Data availability statement

The original contributions presented in the study are included in the article/Supplementary material, further inquiries can be directed to the corresponding authors.

## Author contributions

Conceptualization, writing—review and editing, supervision, and project administration: LH and QD. Methodology: LH, QD, and ZY. Software and formal analysis: ZY and XL. Validation: ZY, MN, and ZL. Investigation: XD and XL. Resources: MN. Data curation: ZL. Writing—original draft preparation: ZY and MN. Visualization: JX. Funding acquisition: LH. All authors read and agreed to the published version of the manuscript.
