# Supplementary material for: Processed meat, red meat, white meat, and digestive tract cancers: A two-sample Mendelian randomization study
Source: Front Nutr. 2023 Feb 13;10:1078963. doi: 10.3389/fnut.2023.1078963 (PMC9968810; doi:10.3389/fnut.2023.1078963)

**Supplementary Figures**

**Processed meat, red meat, white meat and digestive tract cancers: A Mendelian randomization study**

**Figure S1.** Radial plot identifies two outliers in Mendelian randomization analysis from pork intake to colorectal cancer. Ratio estimate for each instrument are shown in the inner radial curve and the overall inverse variance weighting is shown in the outer black radial curve. Blue dots indicate valid genetic instruments. The brown dots represent outliers. IVW: inverse-variance weighted.


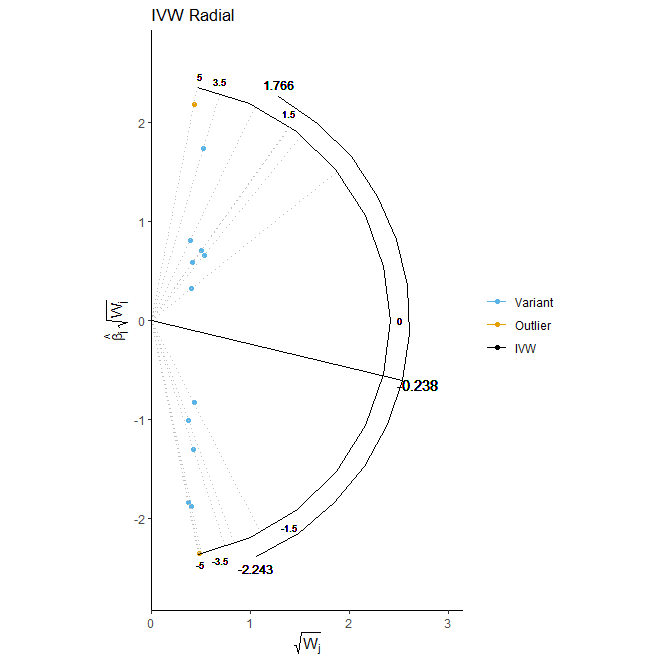


**Figure S2.** Scatter plots, funnel plots, leave-one-out plots from genetically predicted meat intake on esophageal cancer. Scatter plot (A), Leave-one-out plot (B), funnel plot (C) from genetically predicted beef intake on esophageal cancer. Leave-one-out plot (D), scatter plot (E), funnel plot (F) from genetically predicted poultry intake on esophageal cancer. Scatter plot (G), leave-one-out plot (H), funnel plot (I) from genetically predicted lamb intake on esophageal cancer. Scatter plot (J), leave-one-out plot (K), funnel plot (L) from genetically predicted pork intake on esophageal cancer. Scatter plot (M), funnel plot (N), Leave-one-out plot (O) from genetically predicted process meat intake on esophageal cancer.

**
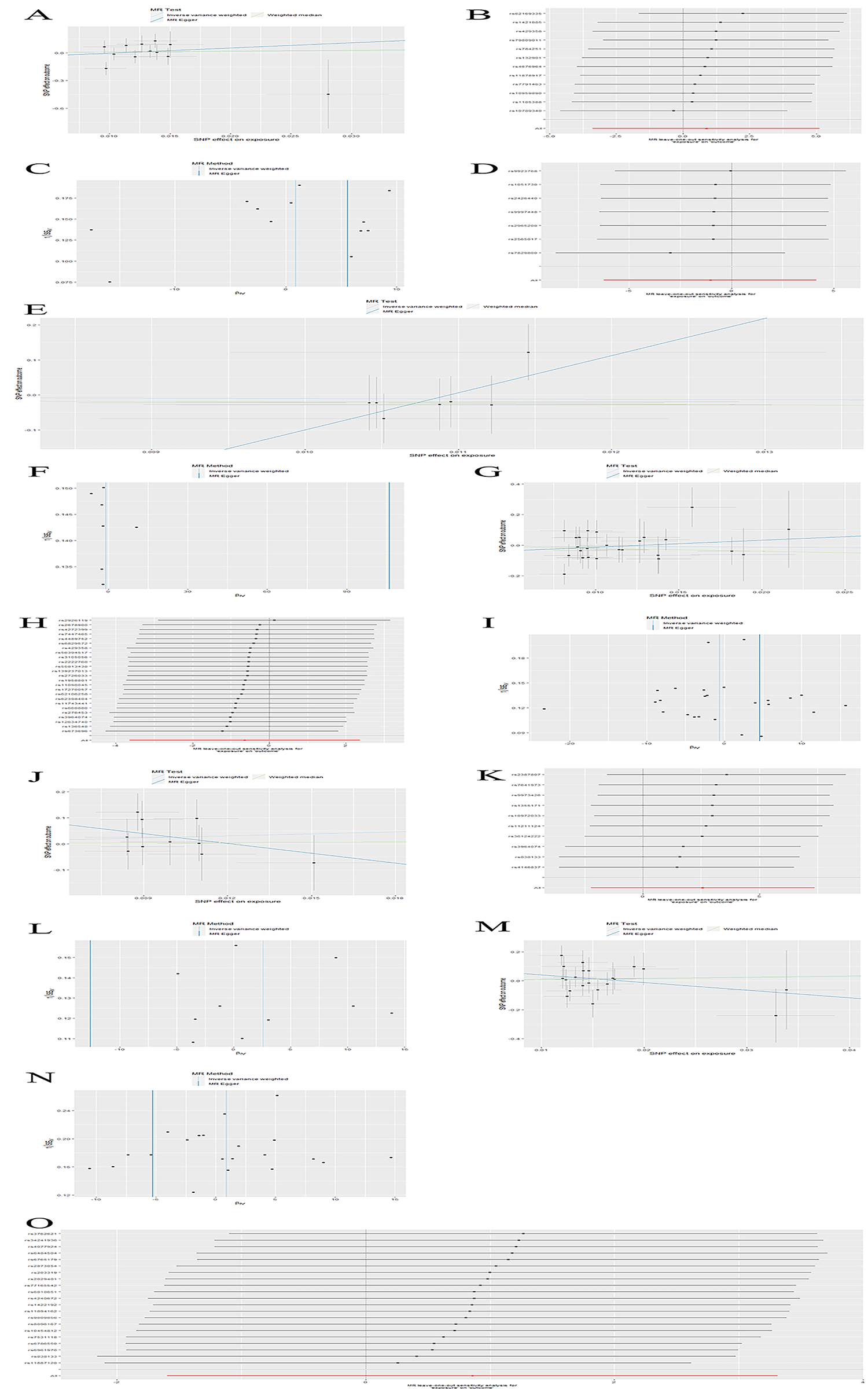
**

**Figure S3.** Scatter plots, funnel plots, leave-one-out plots from genetically predicted meat intake on stomach cancer. Scatter plot (A), leave-one-out plot (B), funnel plot (C) from genetically predicted beef intake on stomach cancer. Scatter plot (D), leave-one-out plot (E), funnel plot (F) from genetically predicted poultry intake on stomach cancer. Scatter plot (G), leave-one-out plot (H), funnel plot (I) from genetically predicted lamb intake on stomach cancer. Scatter plot (J), leave-one-out plot (K), funnel plot (L) from genetically predicted pork intake on stomach cancer. Scatter plot (M), leave-one-out plot (N), funnel plot (O) from genetically predicted process meat intake on stomach cancer.

**
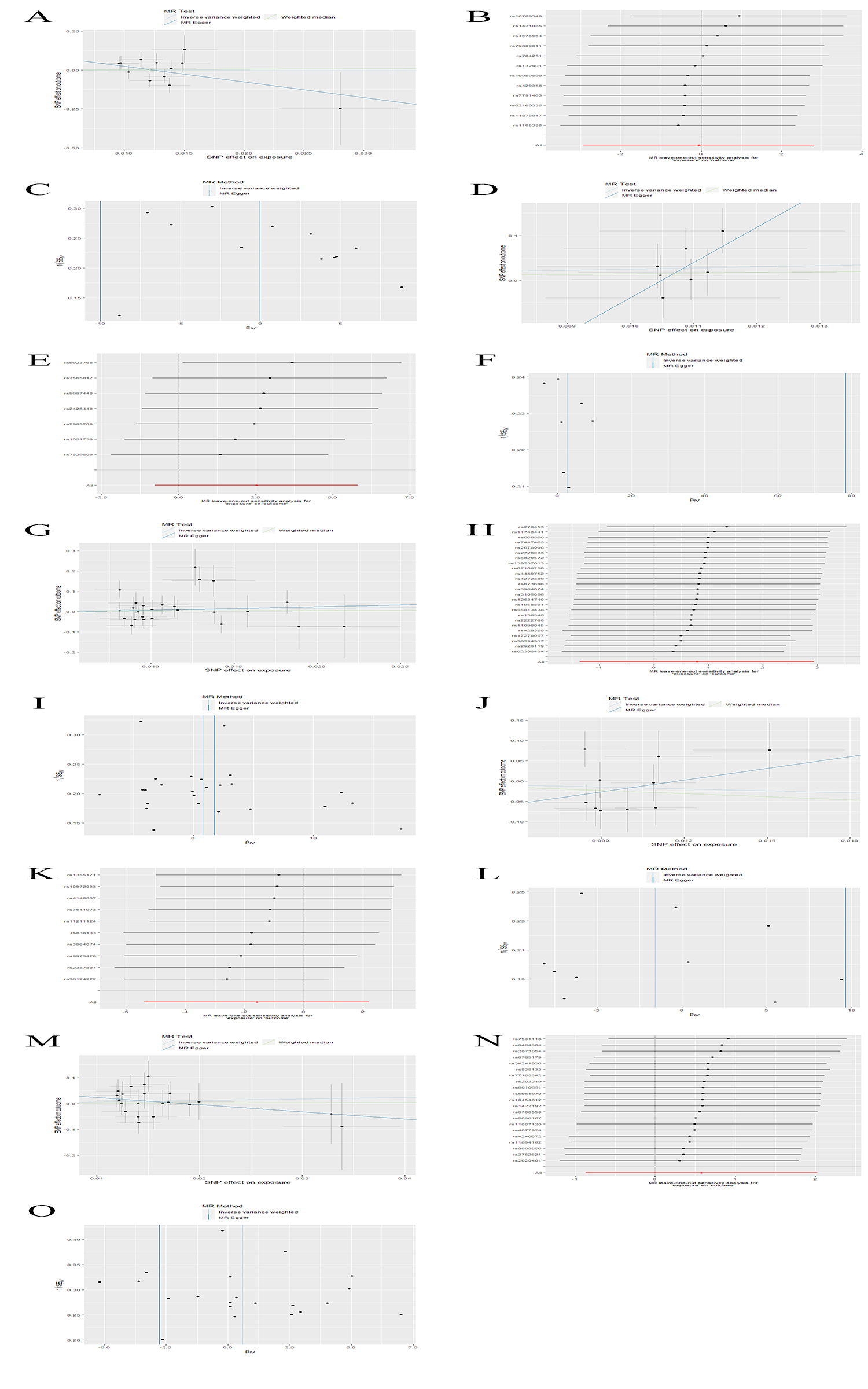
**

**Figure S4.** Scatter plots, funnel plots, leave-one-out plots from genetically predicted meat intake on liver cancer. Scatter plot (A), leave-one-out plot (B), funnel plot (C) from genetically predicted beef intake on liver cancer. Scatter plot (D), leave-one-out plot (E), funnel plot (F) from genetically predicted poultry intake on liver cancer. Scatter plot (G), leave-one-out plot (H), funnel plot (I) from genetically predicted lamb intake on liver cancer. Scatter plot (J), leave-one-out plot (K), funnel plot (L) from genetically predicted pork intake on liver cancer. Scatter plot (M), leave-one-out plot (N), funnel plot (O) from genetically predicted process meat intake on liver cancer.

**
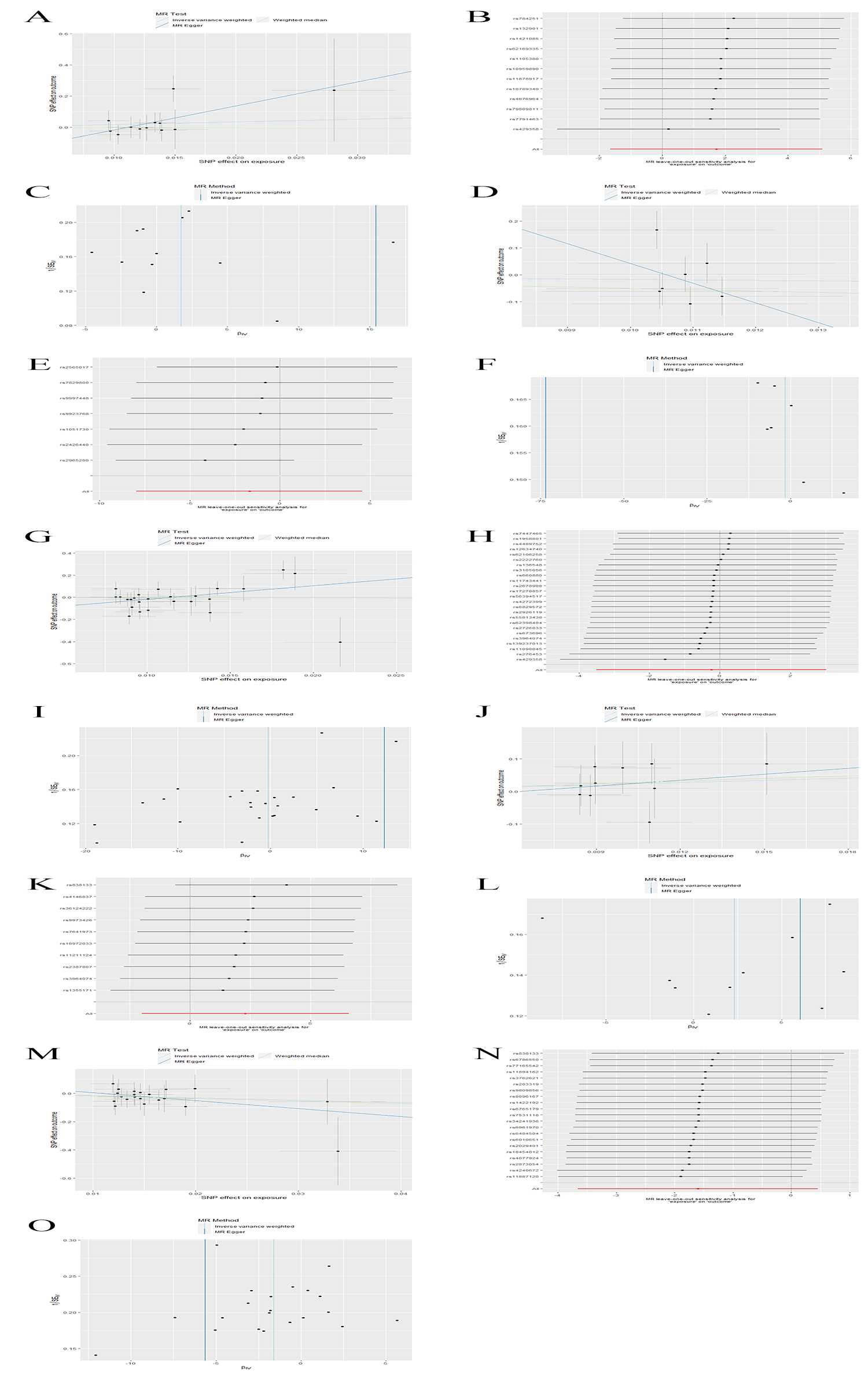
**

**Figure S5.** Scatter plots, funnel plots, leave-one-out plots from genetically predicted meat intake on biliary tract cancer. Scatter plot (A), leave-one-out plot (B), funnel plot (C) from genetically predicted beef intake on biliary tract cancer. Scatter plot (D), leave-one-out plot (E), funnel plot (F) from genetically predicted poultry intake on biliary tract cancer. Scatter plot (G), leave-one-out plot (H), funnel plot (I) from genetically predicted lamb intake on biliary tract cancer. Scatter plot (J), leave-one-out plot (K), funnel plots (L) from genetically predicted pork intake on biliary tract cancer. Scatter plot (M), leave-one-out plot (N), funnel plot (O) from genetically predicted process meat intake on biliary tract cancer.

**
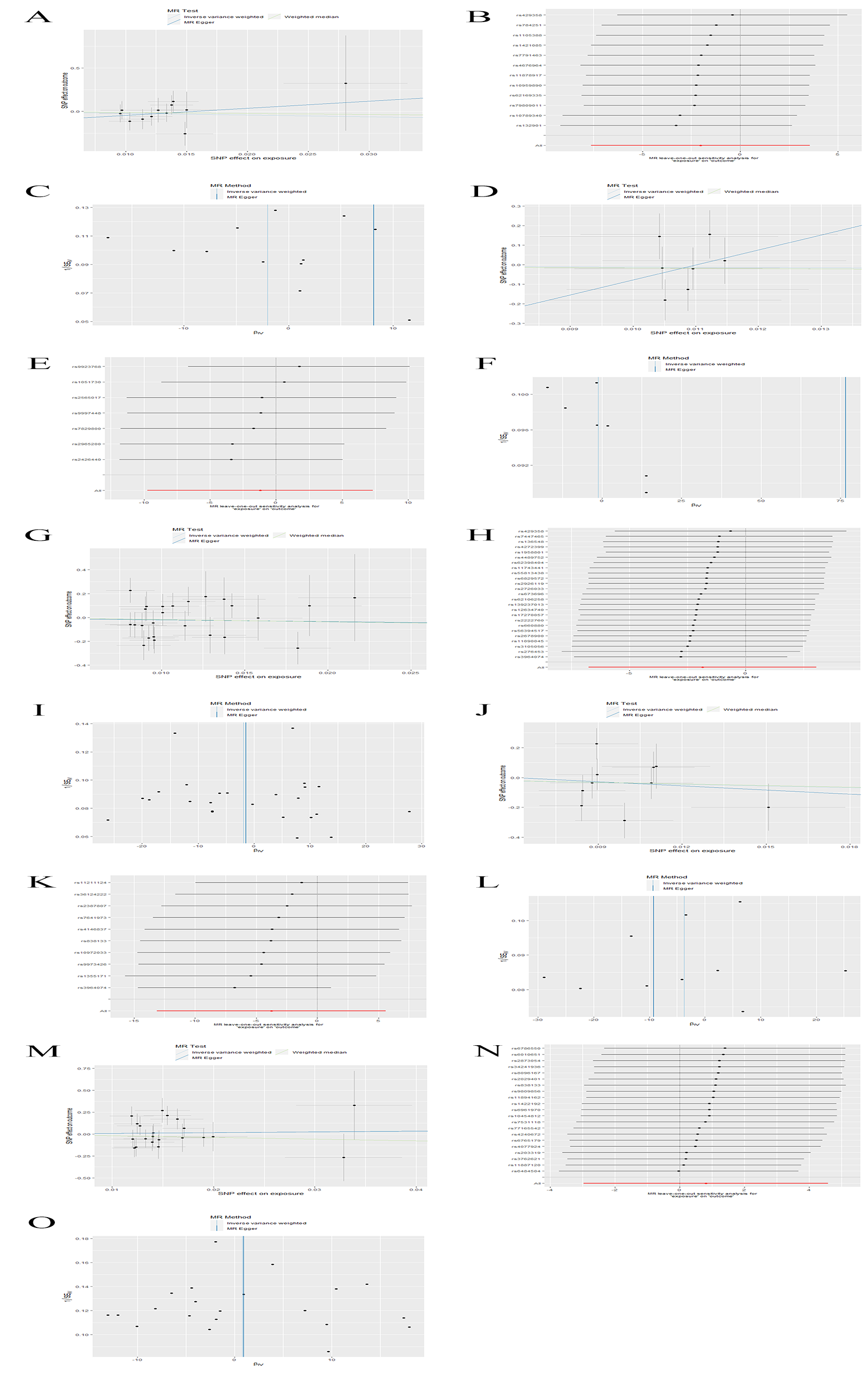
**

**Figure S6.** Scatter plots, funnel plots, leave-one-out plots from genetically predicted meat intake on pancreatic cancer. Scatter plot (A), leave-one-out plot (B), funnel plot (C) from genetically predicted beef intake on pancreatic cancer. Scatter plot (D), leave-one-out plot (E), funnel plot (F) from genetically predicted poultry intake on pancreatic cancer. Scatter plot (G), leave-one-out plot (H), funnel plot (I) from genetically predicted lamb intake on pancreatic cancer. Scatter plot (J), leave-one-out plot (K), funnel plot (L) from genetically predicted pork intake on pancreatic cancer. Scatter plot (M), leave-one-out plot (N), funnel plot (O) from genetically predicted process meat intake on pancreatic cancer.


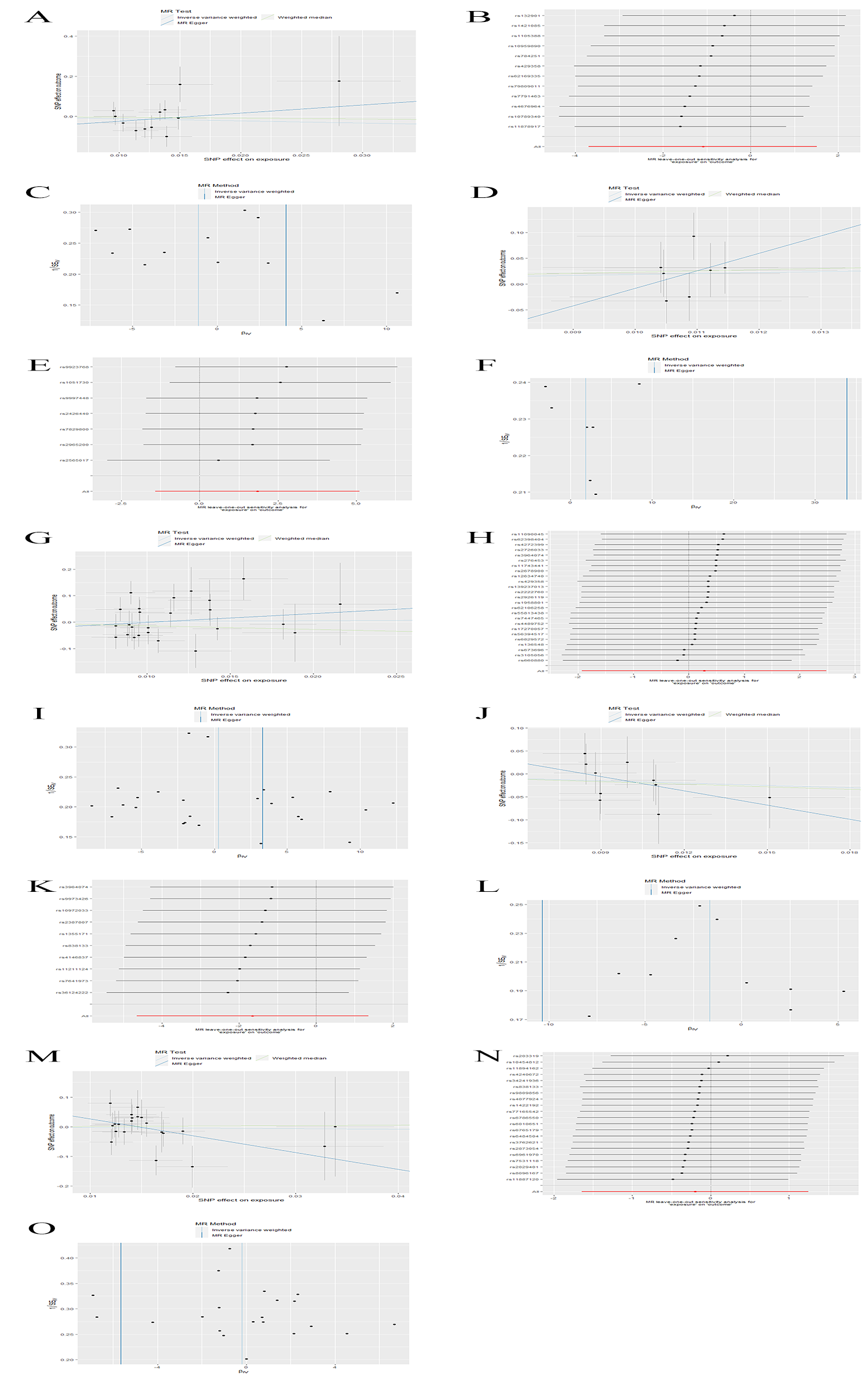


**Figure S7.** Scatter plots, funnel plots, leave-one-out plots from genetically predicted meat intake on colorectal cancer. Scatter plot (A), leave-one-out plot (B), funnel plot (C) from genetically predicted beef intake on colorectal cancer. Scatter plot (D), leave-one-out plot (E), funnel plot (F) from genetically predicted poultry intake on colorectal cancer.


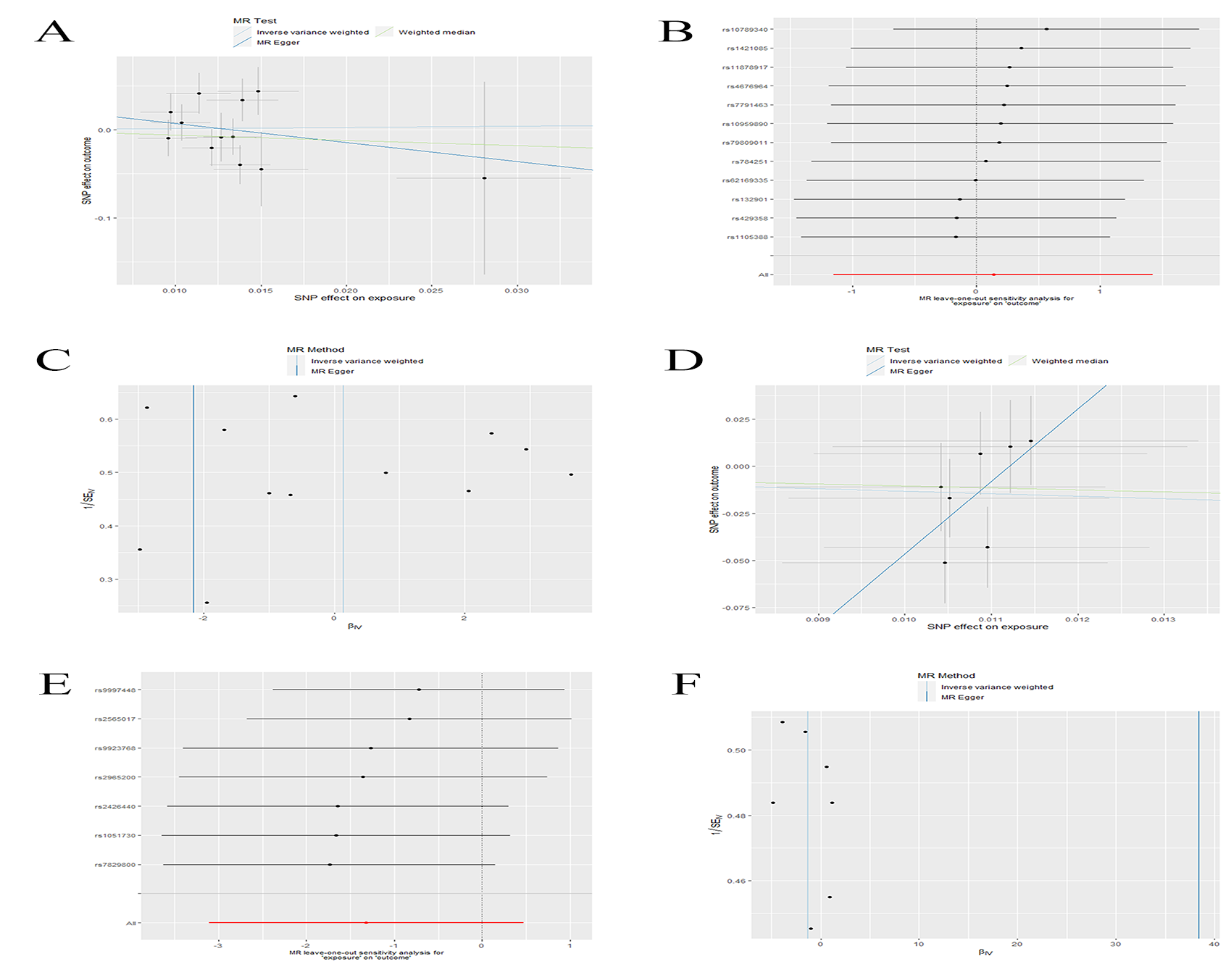


**Figure S8** Scatter plots, funnel plots, leave-one-out plots from genetically predicted meat intake on colorectal cancer. Scatter plot (A), leave-one-out plot (B), funnel plot (C) from genetically predicted lamb intake on colorectal cancer. Scatter plot (D), leave-one-out plot (E), funnel plot (F) from genetically predicted pork intake on colorectal cancer.


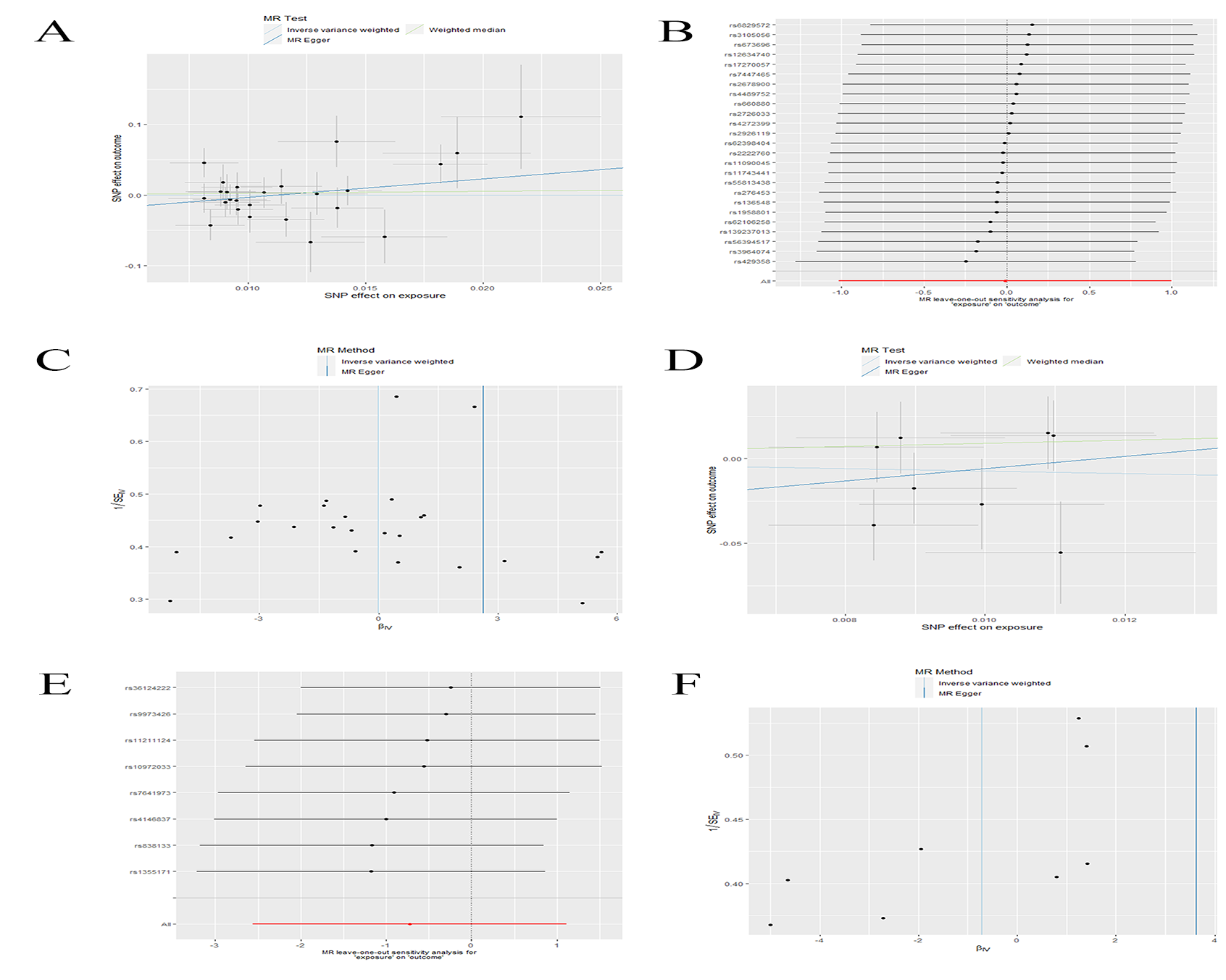


**Figure S9** Scatter plot(A), leave-one-out plot(B), funnel plots (C) from genetically predicted process meat intake on colorectal cancer.

**
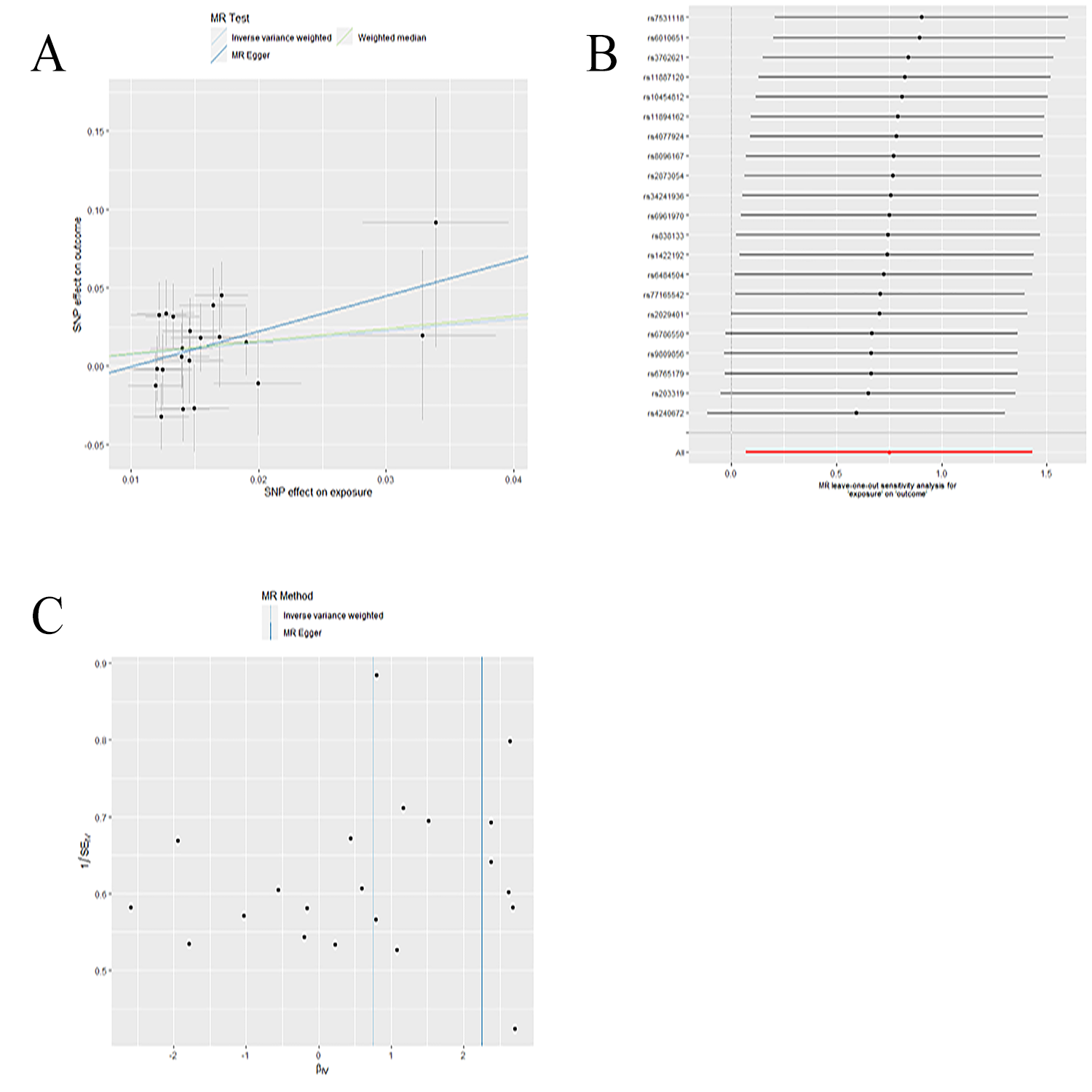
**

**Figure S10** Scatter plot (A), leave-one-out plot (B), funnel plot (C) from genetically predicted process meat intake on colorectal cancer.

**
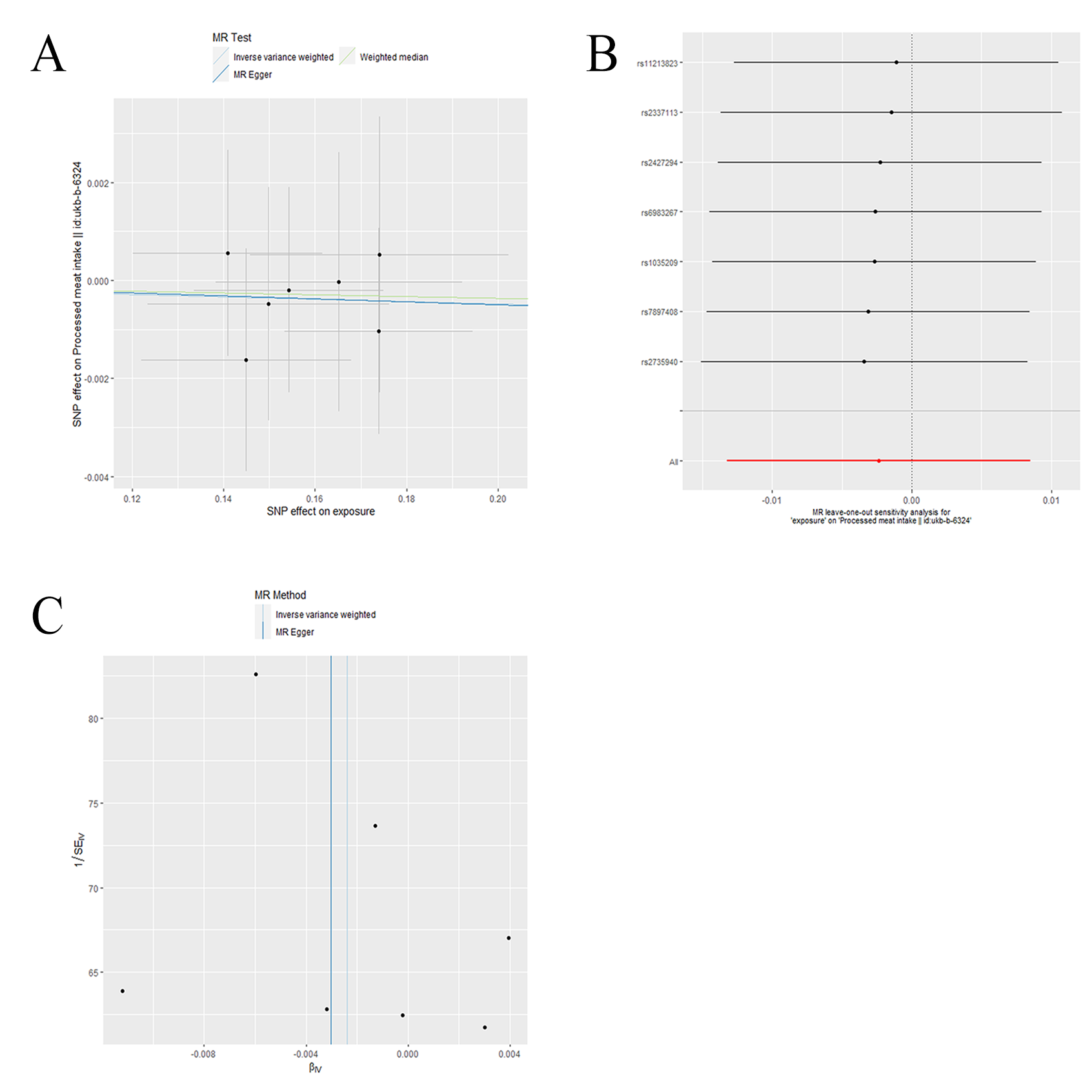
**

**Figure S11** Radial plots, scatter plots, funnel plots, leave-one-out plots from genetically predicted colorectal cancer on BMI and TC. Radial plot (A) identifies 8 outliers in Mendelian randomization analysis from colorectal cancer to BMI. Scatter plot (B), funnel plot (C), leave-one-out plot (D) from genetically predicted p colorectal cancer to BMI. Radial plot (E) identifies 10 outliers in Mendelian randomization analysis from colorectal cancer to BMI. Scatter plot (F), funnel plot (G), leave-one-out plot (H) from genetically predicted p colorectal cancer to TC. Ratio estimate for each instrument are shown in the inner radial curve and the overall inverse variance weighting is shown in the outer black radial curve. Blue dots indicate valid genetic instruments. The brown dots represent outliers. IVW: inverse-variance weighted. BMI: body mass index. TC: total cholesterol.


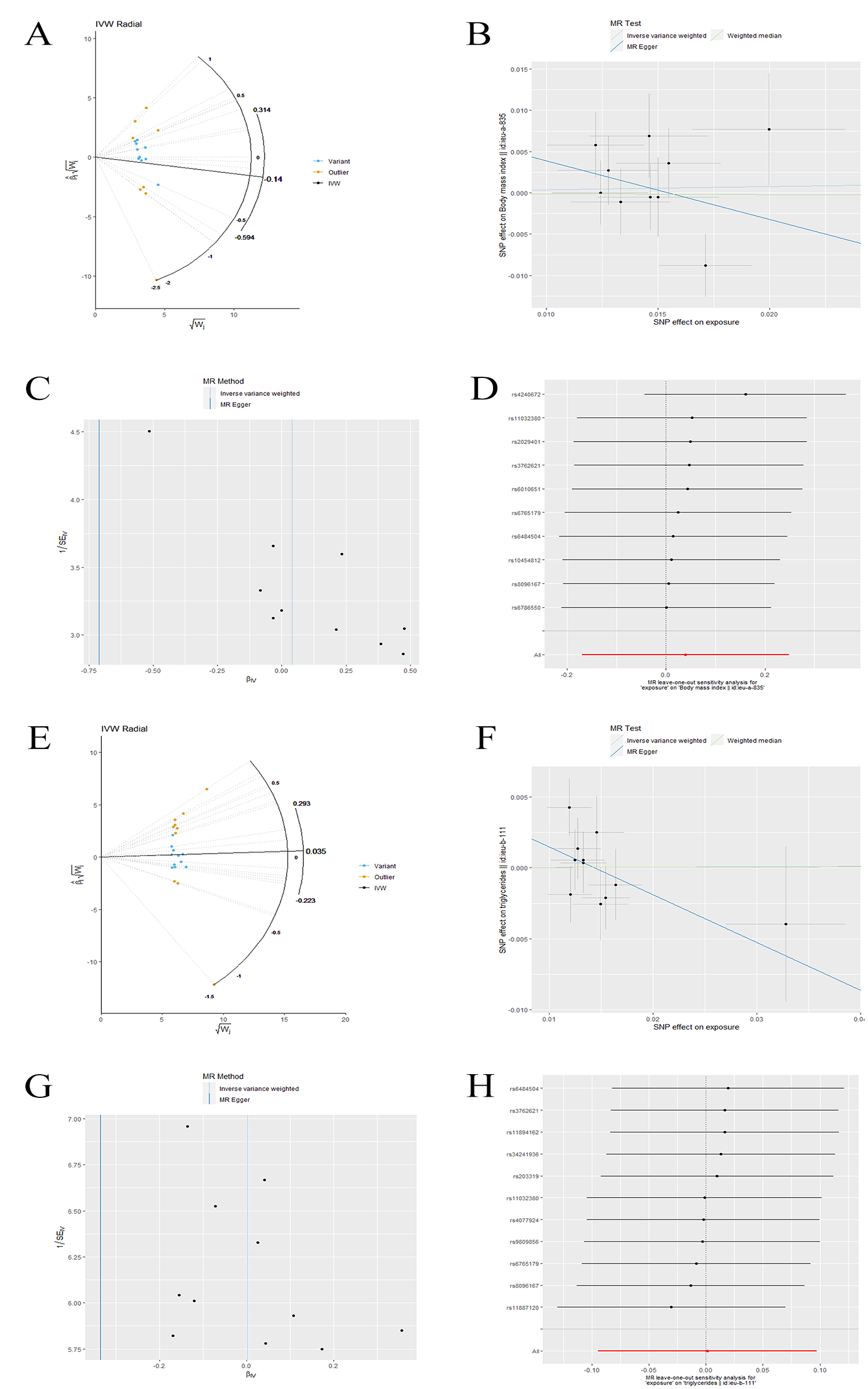

Supplement: Supplementary file 1 [file Data_Sheet_1.docx]
